# What do we know about illness stages in anorexia nervosa?

**DOI:** 10.1192/j.eurpsy.2025.171

**Published:** 2025-08-26

**Authors:** U. Schmidt

**Affiliations:** Psychological Medicine, King’s College London, London, United Kingdom

## Abstract

**Abstract:**

Clinical stageing models are well-established in many areas of medicine, most notably in oncology. These models are used to determine personalised treatment options and indicate prognosis. In psychiatry, staging models have been developed most prominently for psychosis and mood disorders. These models are being used to guide treatment and service provision, e.g. through development of high-risk and early intervention services and stage-appropriate treatment delivery. Clinical staging models for obesity, incorporating medical history, clinical and functional assessments and routine diagnostic investigations also exist and appear to show improved clinical utility in assessing obesity- related risk and prioritising treatment. Recent generic frameworks for staging in mental health have highlighted that there is much individual variability in people’s progression through different illness stages, and therefore there is a need to also specify stage modifiers (disease progression factors (e.g. cognitive or neural factors) and extension factors (e.g. physical complications)). Several staging models for eating disorders have been developed. Most of these focus on anorexia nervosa (AN). Criteria for stage differentiation are mostly based on combinations of cognitive, behavioural and physcial features, and illness duration. As yet most of these models are untested. In my talk I will summarise the available evidence on illness stages, stage modifiers and extension factors and their utility in AN and present evidence on what is known about stage-appropriate interventions. I will discuss implications for research and clinical practice.

**Disclosure of Interest:**

None Declared

